# A Green Approach towards Native Collagen Scaffolds: Environmental and Physicochemical Assessment

**DOI:** 10.3390/polym12071597

**Published:** 2020-07-18

**Authors:** Mireia Andonegi, Ainhoa Irastorza, Ander Izeta, Sara Cabezudo, Koro de la Caba, Pedro Guerrero

**Affiliations:** 1 BIOMAT Research Group, University of the Basque Country (UPV/EHU), Escuela de Ingeniería de Gipuzkoa, Plaza de Europa 1, 20018 Donostia-San Sebastián, Spain; mireia.andonegui@ehu.eus (M.A.); sara.cabezudo@ehu.eus (S.C.); 2 Biodonostia Health Research Institute, Tissue Engineering Group, Pº Dr. Beguiristain s/n, 20014 Donostia-San Sebastián, Spain; ainhoa.irastorzal@ehu.eus (A.I.); ander.izeta@biodonostia.org (A.I.)

**Keywords:** native collagen, chitosan, scaffolds, environmental assessment, degradation

## Abstract

Native collagen scaffolds were prepared in this work, in which both materials and environmental approaches were considered with the aim of providing a global strategy towards more sustainable biomaterials. From the environmental perspective, it is worth mentioning that acid and enzymatic treatments have been avoided to extract collagen, allowing the reduction in the use of resources, in terms of chemicals, energy, and time, and leading to a low environmental load of this step in all the impact categories under analysis. With the incorporation of chitosan into the scaffold-forming formulations, physical interactions occurred between collagen and chitosan, but the native collagen structure was preserved, as observed by Fourier transform infrared (FTIR) and X-ray diffraction (XRD) analyses. The incorporation of chitosan also led to more homogenous porous microstructures, with higher elastic moduli and compression resistance for both dry and hydrated scaffolds. Furthermore, hydrated scaffolds preserved their size and shape after some compression cycles.

## 1. Introduction

Environmental concerns have brought a special interest in natural and renewable materials in terms of sustainability [1,2]. In this regard, the closing-the-loop model promotes the adoption of alternative production systems to increase the resources efficiency by means of a lower consumption of materials and energy in the production scheme, as well as limited discharges into the environment, enabling resources to increase global efficiency. This transition to circular economy provides the opportunity for a more sustainable, efficient, and competitive economy [3]. In this context, by-products can be revalorized and used with the aim of reverting into a social benefit. Green chemistry involves the utilization of eco-friendly and safe materials and processes to decrease the employment of toxic and hazardous substances, using less energy, benign components, and reducing waste products generated during the extraction and manufacture procedures. Hence, the essential aim of green methodologies and technologies is to lessen the harmful results of contaminants on the environment and on living beings. Besides the potential positive impacts of environmentally-friendly products and processes, there are several issues, such as more expensive costs or the lack of knowledge about the performance of some materials, which are still a challenge that must be addressed in the field of cleaner materials for biomedical applications.

Despite such potential, regenerative medicine seems to be the last field to embrace green or environmentally-friendly, processes, since many traditional tissue engineering materials employ organic solvents and synthetic polymers. Considering that one of the essential requirements for the materials used in medicine is the production of biocompatible and biodegradable scaffolds with suitable geometric structures and mechanical properties, mainly animal proteins, such as collagen and gelatin, but some plant proteins have also been explored [4]. Proteins complexity provides these materials with many possibilities to be converted into scaffolds, micro/nanoparticles, fibers, or porous structures with the properties required for medical applications, such as tissue engineering or drug delivery. Most importantly, these biopolymeric networks are stable due to the intra- and inter-molecular bonds formed in the protein structure.

Additionally, some by-products, such as skin remnant from bovine leather tanning industry, have become a collagen source within the green strategy [5,6]. However, not only raw materials but also the processes followed to extract valuable polymers from those by-products are essential in order to move towards more sustainable productive systems. In this regard, the most common methods used to extract collagen include acid and enzymatic treatments, which are harsh and long processes that involve the use of chemicals and energy [7,8]. It is worth noting that those processes have been avoided in this work.

In this context, the environmental assessment is a valuable tool to estimate to which extent certain alternatives based on the valorization of natural and renewable materials could benefit the environmental profile of the entire system, considering both products and processes. Previous works have assessed the environmental impact of leather production [9], more sustainable processes to manufacture leather [10,11,12], or the valorization of leather by-products [13]. However, to the best of our knowledge, there is no report related to the environmental analysis of collagen scaffolds from bovine skins. Therefore, the environmental assessment of collagen scaffolds is carried out in this work with the aim of providing alternative and sustainable sources of biomaterials, a research area that represents one of the challenges to be faced in the field of regenerative medicine.

Because of the inherent properties of proteins, increasing attempts are being made to probe the possibilities of developing biomaterials based on proteins, including micro and nanofibers, hydrogels, porous, and micro and nanoparticles for tissue engineering applications. This interdisciplinary field applies the principles of engineering and life sciences toward the development of biological substitutes, combining cells, biomaterials, and suitable biological factors that restore, maintain, or improve tissue or the whole organ function [14]. In this context, scaffolds play an important role, since they constitute the 3D platforms that provide growing surfaces and stimulate the molecular response of specific cells [15]. Besides promoting cell adhesion and proliferation, biomaterials for tissue engineering applications must meet other requirements, such as integration with the native tissue, a degradation rate proportional to the new tissue formation, mechanical properties suitable for their specific application, chemical stability to support the process of sterilization and storage, and high porosity to provide a proper environment for the extracellular matrix (ECM) secretion and nutrients to cells, all without causing an immune response [16,17]. Therefore, selecting the adequate composition of the scaffold is essential to achieve the optimal environment for cell proliferation and the replacement of the damaged tissue.

In the last few years, various biopolymers, such as proteins and polysaccharides, have been widely investigated and used as alternative biomaterials [18]. Blending with other polymers and crosslinking are some of the typical methods applied to enhance mechanical properties, water resistance, biocompatibility, and the biological performance of these materials in order to make them suitable for biomedical applications. These approaches also reduce the use of traditional non-environmentally friendly tissue engineering polymers. Among them, collagen has been considered as a valuable candidate for regenerative medicine, due to its remarkable capacity to act as a substrate for cell growth, supporting cell attachment, migration, and proliferation [19,20]. Additionally, collagen is biocompatible, biodegradable, and non-immunogenic and, thus, an attractive resource for the formation of scaffolds [21]. Although its fast degradation rate and low mechanical strength limit the use of collagen, its combination with other biopolymers, such as chitosan, can be a strategy to achieve the required properties. In that way, chitosan offers high tensile strength, contains cell adhesion sequences, and improves the functions of fibroblasts, macrophages, and inflammatory cells [22]. Furthermore, chitosan presents other beneficial properties for tissue engineering, such as biocompatibility, biodegradability, non-toxicity, antimicrobial, and antifungal effects [23]. In this regard, some works have reported the effect of crosslinkers on chitosan/collagen scaffolds [24] or the potential of chitosan/collagen scaffolds prepared from collagen extracted from mice tails for periodontal tissue engineering [25].

However, obtaining biocompatible scaffolds with good mechanical properties is still one of the most challenging issues in tissue engineering. In order to have excellent in vitro and in vivo performance, scaffolds should have similar mechanical and biological properties to the natural extracellular matrix. In this context, this work is focused on the development of collagen scaffolds and their assessment from an environmental perspective, avoiding acid and enzymatic treatments with the aim of optimizing the use of resources, as well as on the assessment from a physicochemical perspective, avoiding the use of crosslinkers, such as aldehydes, which can result in being toxic.

## 2. Materials and Methods

### 2.1. Materials

Bovine collagen was supplied by Tenerias Omega (Villatuerta, Spain). Low molecular weight (LMW, 190 kDa) chitosan (batch MKBB9037) and high molecular weight (HMW, 375 kDa) chitosan (batch MMBC0059) with a degree of deacetylation higher than 75% were provided by Sigma-Aldrich (Madrid, Spain). Glycerol (analytical grade, purity of 99.01%) was provided by OPPAC Products (Madrid, Spain), and acetic acid was obtained from Panreac (Barcelona, Spain). 

### 2.2. Preparation of Collagen Scaffolds

The treatment followed to obtain native collagen was described in a previous work [26]. Collagen scaffolds with 30 wt. % HMW or LMW chitosan (on collagen dry basis) were prepared by freeze-drying. Hence, chitosan was dissolved in 100 mL of 0.5 M acetic acid under continuous stirring. Then, 5 g of collagen and 20 wt. % glycerol (on collagen dry basis) were added and the blends were maintained under mechanical stirring for 3 h at 125 rpm. Finally, the blends were poured into each well of a 12 multiwell plate (Costar 3513, Corning Incorporated), and the plate was frozen for 24 h at −23 °C and then freeze-dried (Alpha 1-4 LDplus freeze-dryer, CHRIST) for 48 h. Finally, cylinder-shaped chitosan/collagen samples (2.26 cm diameter and 1 cm height) were removed from the wells. Scaffolds were neutralized by immersion into a sodium hydroxide 0.4 M NaOH solution for 15 min and subsequently rinsed in water. In that way, amino groups of chitosan were deprotonated, leading to the disappearance of ionic repulsions and favoring physical crosslinking with collagen.

### 2.3. Environmental Assessment of Collagen Scaffolds

The environmental analysis was carried out according to ISO 14,040 guidelines and recommendations [27]. The software used was SimaPro 9.0.0.30 (PRé Consultants, The Netherlands). The inventory analysis was carried out considering the materials used in the laboratory and the energy consumption regarding the pretreatments and the preparation steps, as well as the transportation of bovine skins (Bergara-Donostia). Data were obtained from Ecoinvent database. The functional unit considered in this study was 5 g of collagen. Based on the inventory data, environmental impacts were evaluated according to the Hierarchist version of ReCiPe 2016, midpoint. The impact categories analyzed were global warming, stratospheric ozone depletion, ionizing radiation, ozone formation (human health), fine particulate matter formation, ozone formation (terrestrial ecosystems), terrestrial acidification, freshwater eutrophication, marine eutrophication, terrestrial ecotoxicity, freshwater ecotoxicity, marine ecotoxicity, human carcinogenic toxicity, human non-carcinogenic toxicity, land use, mineral resource scarcity, fossil resource scarcity, and water consumption.

### 2.4. Characterization of Collagen Scaffolds

#### 2.4.1. Fourier Transform Infrared (FTIR) Spectroscopy

FTIR spectra were carried out on a Nicolet Nexus FTIR spectrometer using a Golden Gate (Specac), horizontal attenuated total reflectance (ATR) crystal (ZnSe). A total of 32 scans were performed at 4 cm^−1^ resolution. Measurements were recorded between 4000 and 800 cm^−1^.

#### 2.4.2. X-ray Diffraction (XRD)

XRD was performed with a diffraction unit PANalytical Xpert PRO, operating at 40 kV and 40 mA. The radiation was generated from a Cu-Kα (λ = 1.5418 Å) source. Diffraction data were collected from 2θ values from 2 to 50°, where θ is the angle of incidence of the X-ray beam on the sample.

#### 2.4.3. X-ray Photoelectron Spectroscopy (XPS)

XPS was performed in a SPECS spectrometer using a monochromatic radiation equipped with Al Kα (1486.6 eV). The binding energy was calibrated by Ag 3d5/2 peak at 368.28 eV. All spectra were recorded at 90° take-off angle. Survey spectra were recorded with 1.0 eV step and 40 eV analyzer pass energy and the high-resolution regions with 0.1 eV step and 20 eV pass energy. All core level spectra were referenced to the C 1s neutral carbon peak at 284.6 eV. Spectra were analyzed using the CasaXPS 2.3.19PR1.0 software, and peak areas were quantified with a Gaussian-Lorentzian fitting procedure.

#### 2.4.4. Scanning Electron Microscopy (SEM)

A S-4800 scanning electron microscope (Hitachi) was used. Samples were mounted on a metal stub with double-side adhesive tape and coated under vacuum with gold, using a JEOL fine-coat ion sputter JFC-1100 (Izasa) in an argon atmosphere, prior to observation. All samples were examined using an accelerating voltage of 10 kV.

#### 2.4.5. Compression Tests

Compression tests of chitosan/collagen scaffolds were performed using a TA.XT plusC Texture Analyzer equipped with a 50 kg load cell. The analysis was carried out using an aluminium cylinder of 50 mm diameter (P/50). The crosshead speed was set at 1 mm·s^−1^ and the activation force was 5 g. All samples were tested at room temperature and load was applied until the specimen was compressed to 80% of its original height. The software used for the analysis was Exponent 7,0,7,0 Before testing, samples were maintained in a climatic chamber at 25 °C and 50% relative humidity for 48 h. For the tests with hydrated scaffolds, samples were immersed into phosphate buffered saline (PBS) until swelling equilibrium (120 min). The compression test of hydrated scaffolds was carried out 4-fold since scaffolds recovered their initial size at the end of each compression.

#### 2.4.6. Degradation Studies

Degradation studies were conducted in order to determine the weight loss due to the hydrolytic and enzymatic degradation. To this end, the material was cut into 8 mm diameter discs and weighed (W_0_). The scaffolds were then washed with 70% ethanol for 30 min and irradiated with UV for 30 min to be sterilized. Following this, samples were exposed to two degradation agents (PBS and collagenase solution in complete medium) and incubated at 37 °C. After 4 days, the samples were removed, freeze-dried, and weighed (W_4_). The degradation degree (DD) was calculated with the following equation:

(1) $$DD\;\left(\%\right)=\frac{W_{0}-W_{4}}{W_{0}}\times 100,$$

Every test was performed in triplicate. The hydrolytic degradation (HDD) was performed submerging the scaffolds into 500 μL of PBS. To assess the enzymatic degradation (EDD) of the biomaterials, 500 μL of a solution composed of culture medium and collagenase D (Roche, Basel, Switzerland) were added at a working concentration of 1 mg/mL.

## 3. Results and Discussion

### 3.1. Environmental Assessment of Collagen Scaffolds

**Figure 1:**
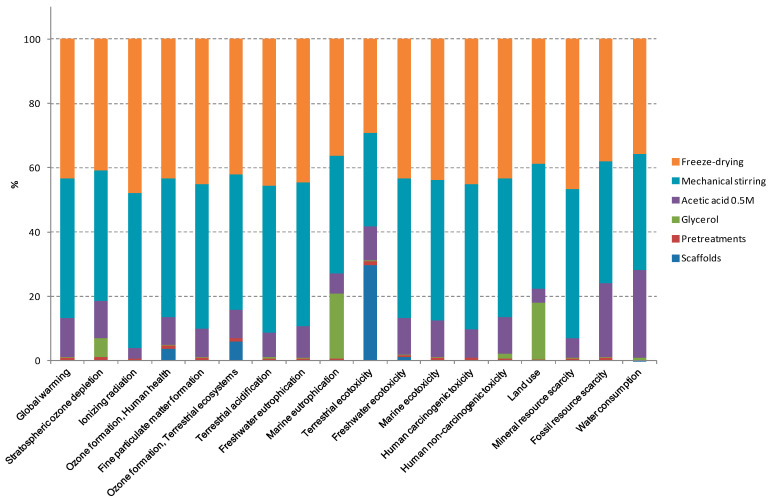
Relative contributions in each impact category for the most relevant processes involved in the entire life cycle of collagen scaffolds. Disaggregating environmental results are displayed in percentage ratios for the most relevant contributing factors: pretreatments, mechanical stirring, acetic acid solution (0.5 M), glycerol, and freeze-drying.

Collagen can be obtained from different animal by-products, such as pig, bovine, and chicken skin and bones, their main sources at commercial scale [28,29]. In this work, collagen was obtained from the trimmings and the splitting-derived by-products [5]. Regarding collagen extraction, it is worth noting that there are several methods reported, which include the pretreatment of raw materials with chemicals, such as sodium hydroxide, butyl alcohol, and acids, or enzymes, such as pepsin and trypsin [30]. In contrast, only sodium hydroxide and mechanical pretreatments were used in this work, reducing the use of chemicals and bringing environmental and economic benefits, since lower amounts of resources (materials, energy, time) were employed in comparison to those works that use acid and enzymatic treatments [7,8]. In the analysis, the use of acetic acid (0.5 M) in a ratio of 1:20 (*w*/*v*) in the scaffolds preparation process, as well as the distilled water production procedure, were also considered. Taken the above into consideration, the global environmental results of collagen scaffolds production are reported in Table 1 for all the impact categories under evaluation. It is worth noting that, since the impact of the pretreatment step in comparison with the scaffolds preparation was so small (Figure 1), the impact values of both processes were jointly represented in Table 1.

Results showed that terrestrial ecotoxicity, global warming, and ionizing radiation caused low environmental damage in the collagen production process, whereas the other categories minimally contributed to the overall environmental burden. Additionally, disaggregating environmental results in percentage ratios are displayed in Figure 1 for each impact category. In this way, the contribution of each stage over the final product can be evaluated. In this case, contributions from the different processes and activities involved throughout the entire life cycle were determined in relation to global results. As can be seen, mechanical stirring and freeze-drying steps were the main contributors to the impact category values, representing around 80% of the total impact. The environmental assessment identified these two processes as the most relevant indicators with higher potential of improvement for decision-making in future works. In particular, the energy used in these processes, specifically electricity consumption, had a critical role in the environmental impact, regardless the impact category considered. Therefore, the assessment indicated that those processes should be improved and optimized in order to reduce the environmental load associated to the development of collagen scaffolds. Since collagen scaffolds were developed at laboratory scale, scaling up processes could lead to achieve the goal of the reduction in the environmental impacts abovementioned.

### 3.2. Fourier Transform Infrared (FTIR) Spectroscopy

**Figure 2:**
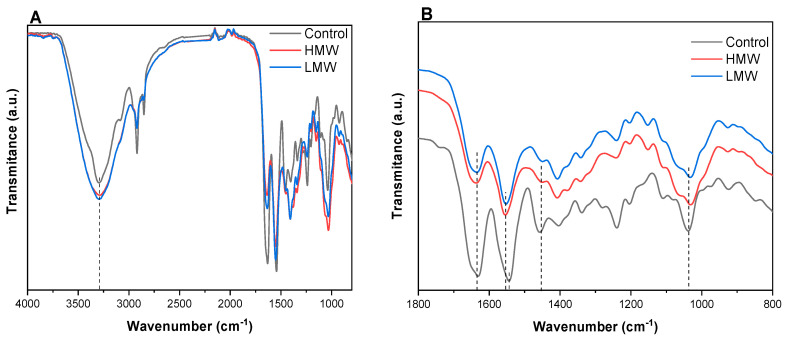
FTIR spectra of control scaffolds and collagen scaffolds with high molecular weight (HMW) and low molecular weight (LMW) chitosan (**A**) from 4000 to 800 cm^−1^ and (**B**) from 1800 to 800 cm^−1^.

FTIR spectra (Figure 2) showed the characteristic absorption bands assigned to the peptide bonds in collagen [31]: 3298 cm^−1^ for amide A (stretching vibrations of N-H groups and O-H), 1631 cm^−1^ for amide I (C = O stretching), 1544 cm^−1^ for amide II (N-H bending), and 1239 cm^−1^ for amide III (C-N stretching). As can be seen in Figure 2A, the band at 3298 cm^−1^ was broadened to some degree when chitosan was incorporated into the scaffold formulation, indicating interactions between collagen and chitosan, both LMW and HMW chitosan. In the same manner, the band at 1038 cm^−1^, associated to C-O vibrations, was notably broadened with the addition of chitosan, as shown in Figure 2B, supporting the interactions between collagen and chitosan. This fact is also in accordance with the change in the relative intensity of the band at 1456 cm^−1^, attributed to CH_2_ bending vibrations of aliphatic groups, which became smaller when chitosan was added. In addition, a change was observed for the relative intensity between amide I and amide II bands.

Additionally, the amide II band shifted towards higher wavenumbers, although the amide I band, related to the collagen triple helix, was maintained at the same position, suggesting that the collagen triple helix could be preserved with the addition of chitosan and that the interactions involved between collagen and chitosan were hydrogen bonds. Furthermore, the wavenumber difference between amide I (V_I_) and amide II (V_II_) bands was lower than 100 cm^−1^, indicating that the triple helix structure of collagen was maintained [32]. As can be seen in Table 2, those values decreased with the addition of chitosan, confirming that chitosan contributed to the preservation of the native collagen structure.

The differences in the relative intensity between FTIR bands, as well as band shifting, suggested physical interactions between collagen and chitosan, probably by hydrogen bonding among carboxyl, amino, and hydroxyl groups present in the components of the scaffold forming formulation.

### 3.3. X-ray Diffraction (XRD)

**Figure 3:**
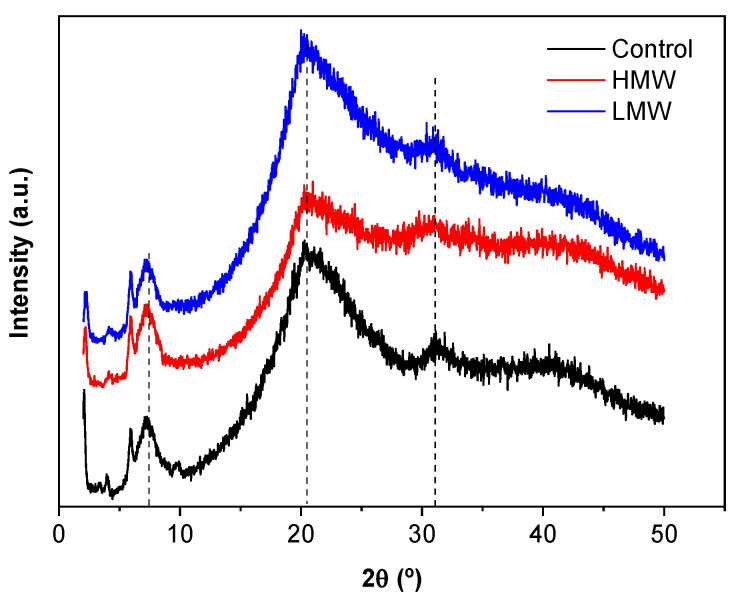
XRD patterns of control scaffolds and collagen scaffolds with HMW and LMW chitosan.

X-ray diffraction (XRD) was carried out to investigate the structure of collagen with LMW and HMW chitosan. As can be seen in Figure 3, the peak around 8° indicated the intermolecular lateral packing distance between the collagen molecular chains, as collagen molecules generally assemble into fibrils by the Schmitt model within chitosan and then formed collagen fibers by intermolecular crosslinking [33]. A second broad peak around 20° resulted from the diffuse scattering of collagen fibers, and the third one around 32° was ascribed to the helical rise per residue distance, related to the conformational integrity of collagen [33]. When chitosan was added, samples showed the same peak pattern, indicating that the structural integrity of collagen was retained, in consistence with FTIR results.

### 3.4. X-ray Photoelectron Spectroscopy (XPS)

**Figure 4:**
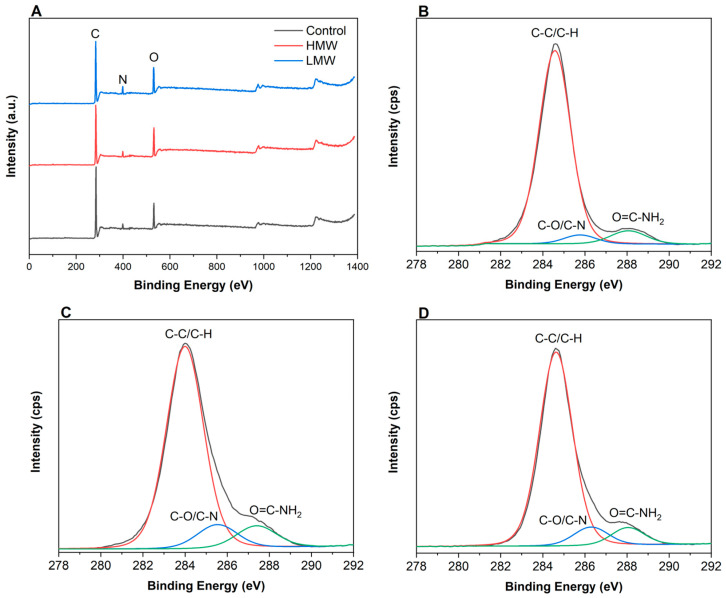
XPS survey spectra of (**A**) all samples and XPS of C 1s features for (**B**) control scaffolds and collagen scaffolds with (**C**) HMW chitosan and (**D**) LMW chitosan.

XPS was performed to get a detailed insight into the corresponding elemental composition. As can be seen in Figure 4A, the predominant peaks identified were those related to C 1s (284.6 eV), N 1s (399.7 eV), and O 1s (531.9 eV) [34]. Furthermore, the C 1s spectra could be fitted into three main separating peaks, at 284.6 eV, 288.1 eV and 285.9 eV. Generally, the peak at 284.6 eV was dominantly attributed to the aliphatic carbons (C-H and C-C), the peak at 285.9 eV was attributed to the carbons associated with oxygen or nitrogen atoms (C-O, C-N), and the peak at 288.1 eV was assigned to carbons in the collagen peptide chain (C = O) [35]. These three relative peak areas of C1s spectra differed from control scaffolds (Figure 4B) and those with HMW (Figure 4C) and LMW (Figure 4D). In particular, the relative area of the peak at 284.6 eV decreased, while those of the peaks at 285.9 and 288.1 eV increased. The decrease of the relative area of the peak corresponding to C-H/C-C bonds, indicated that the hydrophobic character of the surface decreased, while the increase of the relative areas associated to the presence of C-O, C-N, and O = C-NH_2_ suggested that the presence of polar groups towards the surface increased. These facts indicated the increase of the surface hydrophilicity, which improves the bonding capacity of the surface.

As can be seen in Table 3, the peak corresponding to C–O and C–N increased from 3.59% to 6.93% for LMW samples and to 8.18% for HMW samples, may be attributed to the interactions between hydroxyl and amino groups in collagen and chitosan. Additionally, the relative area of the peak associated to the peptide bond increased from 5.89% to 6.21% for scaffolds with LMW and to 7.51% for those with HMW chitosan, probably due to hydrogen bonding among polar groups of collagen and chitosan. Furthermore, the relative areas of O 1s and N 1s were assessed. The weak peak of N 1s appeared at 399.7 eV, and the signal was 1.81% for control scaffolds and increased close to 4% for the scaffolds with chitosan. Additionally, the O 1s spectra was fitted to one peak at 531.9 eV, attributed to O–C=O/O=C–N. This signal represented 12.73% for the control scaffold and increased up to 13.79% and 15.84% for LMW and HMW samples, respectively. This percentage is of great importance since oxygen influences the stability in biological media [36].

### 3.5. Scanning Electron Microscopy (SEM)

**Figure 5:**
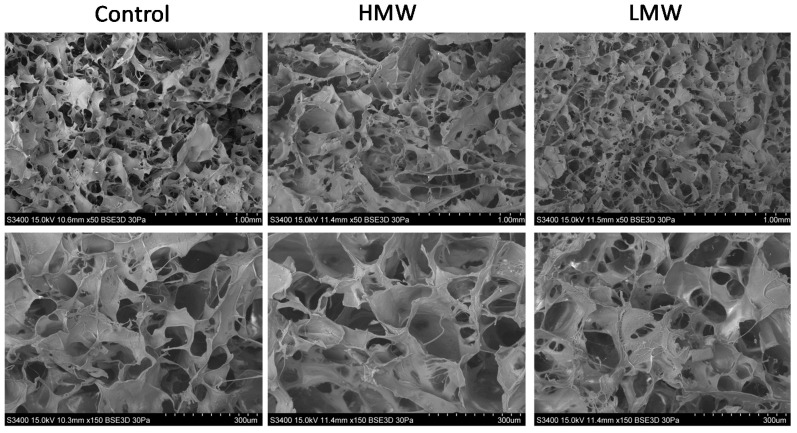
SEM images of control scaffolds and collagen scaffolds with HMW chitosan and LMW chitosan at a magnification of ×50 (**top**) and ×150 (**bottom**).

Since homogenous microporosity is an essential attribute for scaffolds in tissue engineering applications, SEM analysis was carried out to analyze the scaffolds morphology. As can be observed in Figure 5, collagen scaffolds had an evenly distributed three-dimensional reticular pore structure. The LMW group presented a more homogeneous distribution and greater regularity of the pore size as compared to the HMW group. These results would be indicative of the fact that lower molecular weight facilitated the interactions of chitosan with collagen chains, leading to smaller pores. These findings suggest that chitosan/collagen scaffolds have an ideal pore distribution and uniform density for cellular growth in tissue engineering applications [4].

### 3.6. Compression Tests

**Figure 6:**
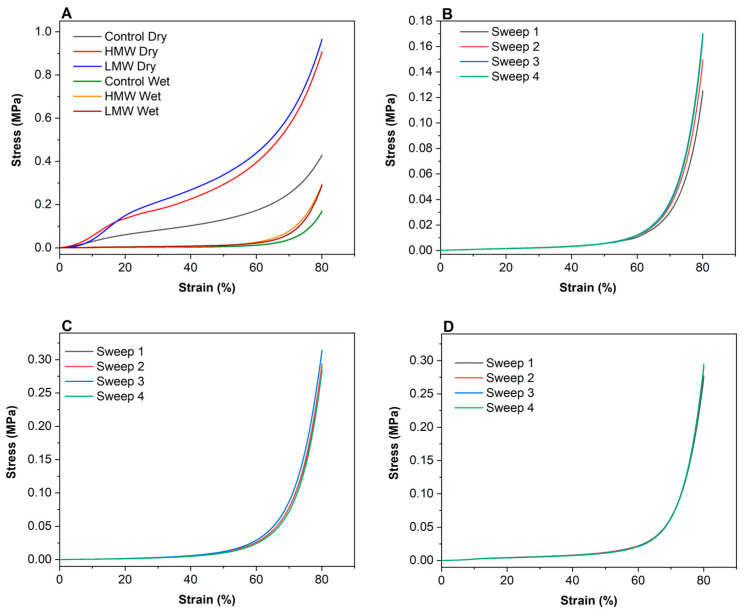
Compression stress–strain curves for (**A**) dry scaffolds, (**B**) hydrated control scaffolds, (**C**) hydrated collagen scaffolds with HMW chitosan, and (**D**) hydrated collagen scaffolds with LMW chitosan.

The required mechanical properties of scaffolds include stretchability, flexibility, and tensile strength in order to provide the ideal 3D growth directing structure to mimic native tissues. Since scaffolds are hydrated in the in vivo environment, compressive properties in the hydrated state are likely to be more relevant and, thus, strength and moduli values were also reported for hydrated collagen scaffolds. As can be seen in Figure 6A, stress-strain curves match the typical compression curves and show elastic and plastic regions. In particular, there were three stages in the stress-strain curves of both dry and hydrated scaffolds: a linear elastic stage (strain < 5%), a steady collapse plateau stage (15% < strain < 50%), and a sharply increasing densification stage (strain > 55%). For biomedical applications, the stress-strain region of larger interest is between 0 and 10% strain; thus, this was the selected section for determining the elastic modulus. 

As expected, there was a notable difference in compressive moduli values between dry and hydrated scaffolds, but, in both cases, the moduli increased with the addition of chitosan, as shown in Table 4. In the same manner, compressive strength showed a remarkable increase when chitosan was incorporated into the scaffold formulation. In this sense, it is worth noting that dry scaffolds did not recover their size after the compression test, while hydrated scaffolds preserved their size under compression. In this regard, the scaffolds with chitosan showed no difference after the four sweeps carried out (Figure 6C,D), while the control scaffolds showed an increase of the stress after each sweep (Figure 6B). This mechanical behavior of collagen scaffolds with chitosan under compression may be explained by the interactions between both biopolymers, as shown by FTIR results.

### 3.7. Degradation Studies

Degradation studies were carried out to evaluate the biomaterial behavior exposed to PBS and collagenase solution (Table 5). Regarding hydrolytic degradation, control scaffolds did not suffer degradation. However, collagen scaffolds with chitosan underwent a degradation of over 10% after 4 days of immersion. The highest HDD value for collagen scaffolds with LMW chitosan may be related to the highest hydrophilicity of these samples, as shown by XPS analysis.

Under the collagenase effect, control scaffolds showed complete degradation, while collagen scaffolds with chitosan showed a degradation around 50% after 4 days of immersion. This may be explained by the fact that collagenase degrades native collagen by targeting the polar zone of the molecule with a (Gly-Pro-y)n sequence, whereas chitosan can only be degraded by enzymes that hydrolyze glucosamine-glucosamine, glucosamine-N-acetyl-glucosamine, and *N*-acetyl-glucosamine-*N*-acetyl-glucosamine bonds, such as chitinases and lysozymes [37]. Therefore, chitosan improved the ability to resist collagenase degradation, preventing the action of the enzyme [38]. The lowest EDD value observed for collagen scaffolds with LMW chitosan may be related to the crosslinking between collagen and chitosan. In this regard, a lower molecular weight facilitates the interactions between collagen and chitosan, leading to a higher crosslinking degree and to a smaller pore size, as observed by SEM; thus, enzymatic degradation is hindered. This study shows the advantages of including chitosan into the scaffold formulation to enhance scaffold biostability, prolonging its biodegradation and reaffirming its potential use for biomedical applications.

## 4. Conclusions

The environmental assessment carried out with the aim of evaluating the environmental impact associated to the main processes involved in the development of collagen scaffolds showed that mechanical stirring and freeze-drying were the processes with a higher environmental load. Although scaling up the production of collagen biomaterials could notably reduce those impacts, further research is needed in order to optimize the conditions used, reducing the energy consumed in those processes. Regarding the physicochemical properties of the scaffolds, FTIR results showed that the interactions between collagen and chitosan were physical interactions by hydrogen bonding, which contributed to stabilize the triple helix structure of native collagen. Additionally, the hydrophilic character of the scaffold surface was enhanced, which is considered a beneficial property in biomaterials in order to promote cell adhesion and proliferation. In this regard, the scaffold morphology presented smaller and more homogenously distributed pores for the scaffolds with LMW chitosan. In addition, these scaffolds showed a more controlled degradation rate under the effect of collagenase, enhancing their biostability.

## Figures and Tables

**Table 1:** Impact category values related to collagen scaffolds.

Impact Category	Unit	Total
Global warming	kg CO_2_ eq	0.7730
Stratospheric ozone depletion	kg CFC11 eq	4.09·10^−7^
Ionizing radiation	kBq Co-60 eq	0.4080
Ozone formation, human health	kg NO_x_ eq	0.0026
Fine particulate matter formation	kg PM_2.5_ eq	0.0019
Ozone formation, terrestrial ecosystems	kg NO_x_ eq	0.0027
Terrestrial acidification	kg SO_2_ eq	0.0048
Freshwater eutrophication	kg P eq	0.0003
Marine eutrophication	kg N eq	3.32·10^−5^
Terrestrial ecotoxicity	kg 1,4-DCB	0.8840
Freshwater ecotoxicity	kg 1,4-DCB	0.0105
Marine ecotoxicity	kg 1,4-DCB	0.0146
Human carcinogenic toxicity	kg 1,4-DCB	0.0233
Human non-carcinogenic toxicity	kg 1,4-DCB	0.3050
Land use	m^2^a crop eq	0.0211
Mineral resource scarcity	kg Cu eq	0.0007
Fossil resource scarcity	kg oil eq	0.2350
Water consumption	m^3^	0.0086

**Table 2:** Wavenumber of amide I (V_I_) and amide II (V_II_) bands, as well as the wavenumber difference between them (V_I_–V_II_), for control scaffolds and collagen scaffolds with HMW and LMW chitosan.

Samples	Amide I (VI)	Amide I (VII)	VI/VII
Control	1631	1544	87
HMW	1636	1555	81
LMW	1635	1552	83

**Table 3:** Area (%) of the XPS spectra peaks for control scaffolds and collagen scaffolds with HMW and LMW chitosan.

Samples	C–C/C–H 284.6 (eV)	C–O/C–N 285.9 (eV)	O=C–NH2 288.1 (eV)	C=N/C–N 399.7 (eV)	O–C=O/O=C–N 531.9 (eV)
Control	75.98	3.59	5.89	1.81	12.73
HMW	69.11	6.93	6.21	3.96	13.79
LMW	64.93	8.18	7.51	3.54	15.84

**Table 4:** Elastic modulus (*E*) and compression strength of control scaffolds and scaffolds with HMW and LMW chitosan.

Samples	E (MPa)		σ (MPa)	
	Dry	Hydrated	Dry	Hydrated
Control	0.122 ± 0.002	0.006 ± 0.001	0.430 ± 0.033	0.170 ± 0.021
HMW	0.563 ± 0.011	0.013 ± 0.003	0.951 ± 0.041	0.294 ± 0.012
LMW	0.423 ± 0.013	0.018 ± 0.004	0.965 ± 0.049	0.291 ± 0.008

**Table 5:** Hydrolytic (HDD) and enzymatic (EDD) degree of degradation for control scaffolds and collagen scaffolds with HMW and LMW chitosan after 4 days.

Samples	HDD (%)	EDD (%)
Control	0	100
HMW	10.6 ± 1.8	55.8 ± 1.6
LMW	15.1 ± 0.4	44.4 ± 4.4
